# Differential Eating Behavior Patterns among the Dark Triad

**DOI:** 10.3390/ijerph19127062

**Published:** 2022-06-09

**Authors:** Liping Shi, Shijin Sun, Yaoguo Geng

**Affiliations:** 1 Department of Psychology, Fudan University, Shanghai 200433, China; 21110730011@m.fudan.edu.cn (L.S.); sunshijin@fudan.edu.cn (S.S.); 2 School of Education, Zhengzhou University, Zhengzhou 450001, China

**Keywords:** Dark Triad, eating behavior, associations

## Abstract

There is little extant empirical literature examining the associations between Dark Triad (DT: Machiavellianism, narcissism, and psychopathy) and eating behaviors. The current study (*n* = 361) investigated the associations between Dark Triad and restrained eating, uncontrolled eating, and emotional eating in a sample drawn from the general population. The results from the study indicate that (a) despite expected sex differences in narcissism and primary psychopathy, no sex differences were found in Machiavellianism, secondary psychopathy, and eating behaviors; (b) among women, Machiavellianism was a protective factor against uncontrolled eating behaviors; (c) the sex of the participant moderated the narcissism–uncontrolled eating behaviors and narcissism–emotional eating behaviors relationships, with the negative correlation being stronger for men than that for women; (d) secondary psychopathy, rather than primary psychopathy, was associated with higher uncontrolled eating behaviors in both sexes, and associated with higher emotional eating behaviors for men only. The implication of these findings are interpreted and discussed.

## 1. Introduction

Eating disorders are defined with special regard to body weight/shape and associated behaviors, such as dieting, binge eating, purging, and excessive exercising. On the basis of body weight, the DSM-5 divides eating disorders into two main types: anorexia nervosa and bulimia nervosa [1].

Clinicians and researchers have suggested that eating disorders are closely associated with personality traits. In a recent review, Farstad and colleagues revealed that higher perfectionism, neuroticism, avoidance motivation, sensitivity to social rewards, and lower extraversion and self-directedness are common in all eating disorder diagnoses [2]. Further, among eating disorders, greater impulsivity is common in bulimia nervosa, avoidance and obsessive–compulsive personality disorders are the most common in restricting anorexia nervosa (one subtype of anorexia nervosa), and borderline and paranoid personality disorders are common in binge eating/purging anorexia nervosa (another subtype of anorexia nervosa), bulimia nervosa, and eating disorders not otherwise specified.

Eating disorders were found to occur almost exclusively in women [3]. In women with eating disorders, empirical studies have identified three personality subtypes: the high functioning/perfectionist, overcontrolled, and dysregulated subtypes [1,4,5]. Among the three, the high-functioning/perfectionist subtype and overcontrolled subtype are associated with either anorexia nervosa or bulimia nervosa, while the dysregulated subtype is most closely associated with bulimia nervosa [1]. Accordingly, it seems that Cluster C personality traits are the most prevalent among those with anorexia nervosa and bulimia nervosa.

Although a certain study involved the association between bulimia nervosa and the personality constructs of narcissism [6], few studies to date have explored the relationship between Dark Triad traits (i.e., Machiavellianism, narcissism, and psychopathy) and eating disorders/behaviors. The Dark Triad refers to a term that was introduced by Paulhus and Williams to describe three overlapping but distinct personality traits that are socially undesirable and comprise malevolent characteristics [7]. Machiavellianism is characterized by a belief in the effectiveness of manipulative tactics in dealing with other people, a cynical view of human nature, and a moral outlook that puts expediency above principle [8,9,10]. Narcissism is marked by exaggeration of self-worth and importance, superiority over others, bragging, attention and admiration seeking, and manipulation [11,12]. Lastly, psychopathy is defined by impulsivity and sensation seeking, callousness, a lack of remorse, and antisocial behaviors [10,11,13]. Research has suggested that lower agreeableness, lower honesty–humility, interpersonal manipulation, and callous affect were the common characteristics shared by the Dark Triad [14,15].

Recently, three important studies addressed the links between Dark Triad and eating behaviors. Specifically, Sariyska and colleagues first underlined the role of Dark Triad in the context of eating style, and found that the group of omnivores scored higher on Machiavellianism, narcissism, and psychopathy than the group of vegans/vegetarians did [16]. In 2020, Mertens and colleagues examined the relationship between Dark Triad, and meat-eating justification and meat consumption in Germany, and noted that Machiavellianism was partly able to explain gender differences in meat-eating justification strategies and behaviors [17]. More recently, Mertens and colleagues examined the relationship between Dark Triad and eating behaviors in a large sample of German population, and also found evidence that Machiavellianism plays an important role in explaining gender differences in meat-eating justification strategies [18].

Although the works of Sariyska et al., and Mertens et al. are influential in establishing associations between Dark Triad and eating behaviors [16,17,18], the three studies have several limitations. First, they assessed psychopathy globally and did not differentiate the forms of psychopathy (i.e., primary and secondary psychopathy) [10]. Second, participants were all recruited from Germany. So, cross-cultural evidence is needed to confirm the findings. Third, three studies have ignored the links between Dark Triad and disordered eating behaviors (between eating disorders and normal eating style), such as emotional eating.

Given the extent to which each Dark Triad trait influences behaviors [19], given the close associations between Dark Triad and fast life history strategies (a fast life history strategy is reflective of reproductive efforts over somatic efforts and mating efforts over parental effort, and affects various aspects of human psychology, including disordered eating style) [15,20,21], and given the sex differences in eating disorders [3], it seems reasonable to extend previous findings to the links between Dark Triad and disordered eating behaviors. On the basis of theory analysis, the current study aims to gain a deeper understanding of the associations between Dark Triad traits and disordered eating behaviors. We predict that (a) Dark Triad traits are positively associated with disordered eating behaviors and its dimensions; (b) each Dark Triad trait could uniquely contribute to the prediction of eating behaviors, and psychopathy (more specifically secondary psychopathy) would be most closely associated with disordered eating behaviors, such as uncontrolled and emotional eating behaviors; and (c) the sex of the participant could moderate the associations between the Dark Triad and eating behaviors, and these associations are especially strong for women.

## 2. Materials and Methods

### 2.1. Participants

To reach a large number of participants, an online questionnaire was used for data collection. The link to the survey was distributed via several social media platforms in July 2019. After receiving informed consent, participants were assured that their answers were confidential and anonymous. In total, 378 individuals started the online questionnaire; after dropping incomplete and invalid data, 361 respondents remained. The final sample consisted of 248 (68.7%) women and 113 (31.3%) men aged 18–56 (M = 24.83, SD = 7.45). Among these participants, 0.8% had a junior high school degree, 8% had a high-school degree and technical secondary school qualifications, 75.3% had a college degree, 22.4% had a master’s degree, and 2.2% had a doctorate degree. After fulfillment of the research requirement, participants received CNY 10 (approximately USD 1.5).

### 2.2. Materials

#### 2.2.1. Machiavellian Personality Scale (MPS)

MPS is a 16-item, self-rating, and validated measure designed to assess four dimensions of Machiavellianism: (a) amorality (e.g., “I am willing to be unethical if I believe it will help me succeed”), (b) desire for control (e.g., “I like to give the orders in interpersonal situations”), (c) desire for status (e.g., “status is a good sign of success in life”), and (d) distrust of others (e.g., “people are only motivated by personal gain”) [22,23]. Each item was rated on a 5-point Likert scale anchored by 1 (strongly disagree) and 5 (strongly agree). All items were summed to create a total score (range 16–80), and a higher score was indicative of higher levels of Machiavellianism. This scale was previously used among Chinese samples with satisfactory reliability and validity [24]. In this study, Cronbach’s alpha was 0.868 for entire scale, 0.809 for amorality, 0.845 for desire for control, 0.768 for desire for status, and 0.819 for distrust of others. Due to varying factorial structures of Machiavellianism construct [16], we only used the total score.

#### 2.2.2. Narcissistic Personality Inventory-Brief Version (NPI-16)

NPI-16 is a 16-item, self-rating, and validated measure designed to assess individual differences in levels of narcissism, which was validated in the Chinese sample [25,26]. NPI-16 has a dichotomous, forced-choice response format. Each item on the scale presents two statements, one indicative of narcissism and the other not (e.g., A: “I think I am a special person” or B: “I am no better or no worse than most people”). Participants were asked to indicate which best described themselves, scored 1 = narcissistic response, 0 = non-narcissistic response. All items were summed to create a total score (range 0–16), and a higher score was indicative of higher levels of narcissism. In this study, Cronbach’s alpha was 0.818 for entire scale.

#### 2.2.3. Levenson Self-Report Psychopathy Scale (LSRP)

LSRP is a 26-item, self-rating, and validated measure designed to assess two factors: (a) primary psychopathy (e.g., “I enjoy manipulating other people’s feelings”) and (b) secondary psychopathy (e.g., I have been in a lot of shouting matches with other people). Each item was rated on a 4-point Likert scale anchored by 1 (strongly disagree) and 4 (strongly agree) [27]. This scale was previously validated among Chinese samples [28]. The score for each factor is generated by adding the scores of items within that factor, all items are summed to create a total score (range 26–104), and a higher score is indicative of higher levels of psychopathy. In this study, Cronbach’s alpha was 0.857 for the entire scale, 0.811 for primary psychopathy, and 0.730 for secondary psychopathy.

#### 2.2.4. Three Factor Eating Questionnaire-R18 (TFEQ-R18)

TFEQ-R18 is an 18-item, self-rating, and validated measure designed to assess three different aspects of eating behaviors: (a) restrained eating (i.e., conscious restriction of food intake aimed to control body weight and/or to promote weight loss), (b) uncontrolled eating (i.e., the tendency to eat more than usual due to a loss of control over intake with a subjective feeling of hunger, (c) emotional eating (i.e., the inability to resist emotional cues, eating as a response to different negative emotions) [29]. Each item was rated on a 4-point Likert scale anchored by 1 (definitely true) and 4 (definitely false). The score for each factor was generated by adding the scores of items within that factor, *and a higher score was indicative of lower levels of restrained eating*, *uncontrolled eating*, or *emotional eating*. The Chinese version of TFEQ-R18 was obtained by conducting a translation and back-translation, without any overlap across the members who performed the translation and back-translation. The original and back-translated items were compared for nonequivalence of meaning, and discrepancies were revised. The process continued until no semantic differences were noticed between the original version and the Chinese version. In this study, the results of the CFA revealed that the 18-item three-factor model fitted the data well (χ^2^/df = 2.066, RMSEA = 0.054, NFI = 0.931, CFI = 0.963, GFI = 0.924); Cronbach’s alpha was 0.868 for entire scale, 0.847 for restrained eating, 0.900 for uncontrolled eating, and 0.865 for emotional eating.

## 3. Results

### 3.1. Sex Differences

Table 1 shows the means and standard deviations for all variables. As expected, men reported higher levels of narcissism and primary psychopathy than those of women. The sex difference in Machiavellianism was not significant, although men scored marginally higher than women did. As for eating behaviors, the present study failed to find any sex differences in its three factors.

### 3.2. Correlations

There is evidence that the Dark Triad has different predictors in the two sexes [19]; we then separately examined correlations between Dark Triad and eating behaviors for men and women. Results are presented in Table 2. In neither sex was Machiavellianism associated with restrained eating, uncontrolled eating, and emotional eating. For men only, narcissism was negatively and significantly associated with uncontrolled eating and emotional eating. In both sexes, both primary psychopathy and secondary psychopathy were negatively and significantly associated with uncontrolled eating.

When these correlations were assessed across the sexes, only two differed significantly. The correlations between narcissism and uncontrolled eating (*r* = −0.389, *p* < 0.01 for men; *r* = −0.087, *p* > 0.05 for women; Fisher’s z = 2.818, *p* < 0.01), emotional eating (*r* = −0.308, *p* < 0.01 for men; *r* = −0.011, *p* > 0.05 for women; Fisher’s z = 2.678, *p* < 0.01) were stronger in men than those in women, thereby suggesting that the impact of narcissism on uncontrolled eating and emotional eating may differ significantly in two sexes.

### 3.3. Regression Analyses

Multiple regression analyses were performed separately for men (coded = 1) and women (coded = 2) to explore differential eating behavior patterns among Dark Triad traits [30]. We controlled for age and educational degree in the regression analyses by entering them in Step 1, followed by Dark Triad in Step 2. As shown in Table 3, in both sexes, Dark Triad had no correlation with restrained eating. As shown in Table 4, Machiavellianism was associated with lower uncontrolled eating for women only, narcissism was associated with higher uncontrolled eating for men only, and secondary psychopathy was associated with higher uncontrolled eating in both sexes. As shown in Table 5, narcissism and secondary psychopathy were associated with higher emotional eating for men only. In addition, for women only, age was associated with lower uncontrolled eating and emotional eating, and strikingly, higher educational degree was associated with higher levels of emotional eating.

### 3.4. Moderating Effect of Sex

**Figure 1:**
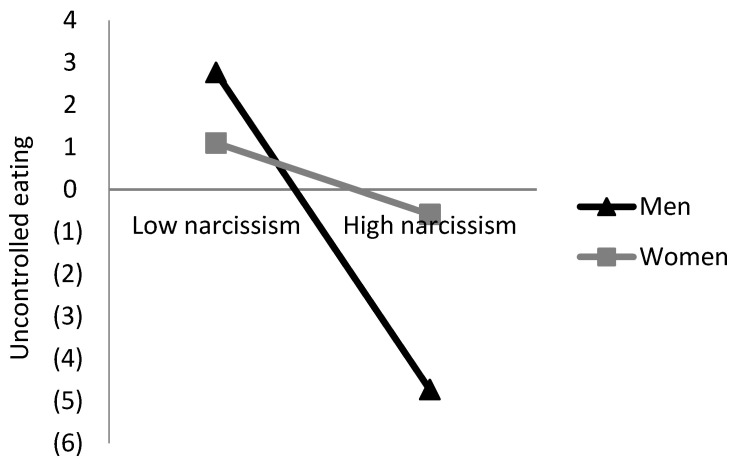
Uncontrolled eating as a function of narcissism and sex.

**Figure 2:**
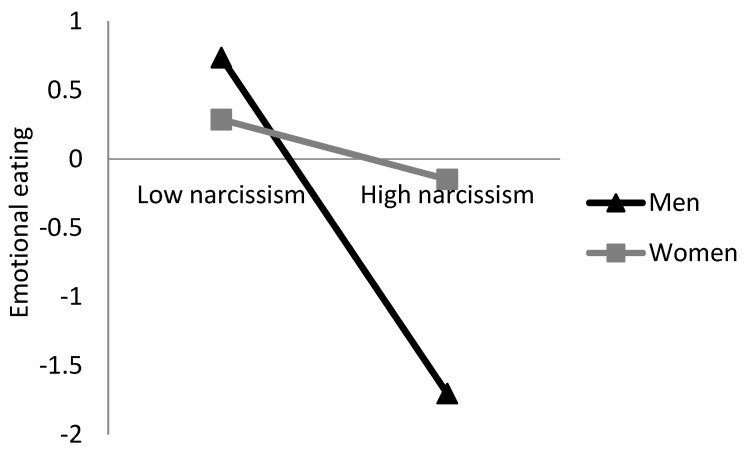
Emotional eating as a function of narcissism and sex.

Because narcissism has different predictors in the two sexes, we conducted formal moderation analysis to confirm whether the sex of the participant moderated the associations between narcissism and eating behaviors [31]. After controlling for age and educational degree, the sex–narcissism interaction term was negatively and significantly associated with uncontrolled eating (*β* = −0.197, *t* = 2.91, *p* < 0.01) and emotional eating (*β* = −0.196, *t* = 2.85, *p* < 0.01). These results mean that the link between narcissism and uncontrolled/emotional eating was more substantial for men than it is for women (Figure 1 and Figure 2).

## 4. Discussion

The Dark Triad is a hot topic in personality psychology, clinical psychology, and evolutionary psychology. Researchers have examined various intrapersonal, interpersonal, and behavioral correlates. In current study, we conducted an exploration study to examine the associations between the Dark Triad and eating behaviors.

Consistent with previous studies [32,33,34], the results of sex differences in the Dark Triad indicate that men scored significantly higher than women did on narcissism and primary psychopathy. Men scored higher than women did in Machiavellianism, but the sex difference was slight and not significant. Additionally, no sex difference was found in three aspects of eating behaviors in present study.

The fact that Machiavellianism was associated with lower uncontrolled eating behaviors deserves attention. Among women, when shared variance between the traits of Dark Triad was controlled in multiple regression, Machiavellianism (*β* = 0.197, *p* < 0.05) uniquely predicted uncontrolled eating, thus suggesting that it was a protective factor against uncontrolled eating behaviors.

Machiavellianism and even Dark Triad, may tap into a fast life strategy [35,36,37,38]. Life history theory is a midlevel evolutionary theory about resource allocation that describes the adaptive choices made by people to optimize survival and reproduction on account of ecological and/or social environments [20]. The fast life history strategy is produced by harsh or unpredictable environments encountered in childhood [20], is reflective of reproductive efforts (an early age of reproduction and a preference for immediate benefits at the expense of long-term benefits) over somatic efforts (people devoted to their own continuing survival and development), and is adaptive under adverse circumstances [21]. For example, an experimental study has shown that information associated with harsh environments encourages behaviors consistent with a fast life history strategy, unconsciously leading participants to seek and consume more filling and high-calorie foods [39] that they believe will sustain them for a long time. Therefore, the relationship between Machiavellianism and relevant eating behaviors is very pertinent in view of the results.

However, some studies found that those high in Machiavellianism have strategic planning and a longer-term orientation [40,41]. Perhaps these characteristics may promote a slow life strategy [42] and thereby diminish uncontrolled eating behaviors. Another possible hypothesis is that dieting may work primarily as a female strategy in mating and status competition [1]. Dieting and the resulting thinness can increase one’s attractiveness and enhance status in female groups, especially when cultural and fashion emphasis on thinness is strong. From an evolutionary perspective, the psychological mechanisms that underlie dieting behaviors are fundamentally adaptive [1].

Both multiple regression and moderation analyses indicate that only men showed a significant narcissism–uncontrolled eating/emotional eating slope, thus demonstrating that the sex of the participant could moderate the simple relationships between narcissism–uncontrolled/emotional eating. These results suggest that narcissism uniquely predicted reckless eating behaviors in men. An explanation for the obtained results is that narcissism is positively associated with impulsivity. For example, Crysel and colleagues found that, of the Dark Triad traits, narcissism was most consistently associated with behavioral risk tasks, and may be driving the observed relationships between the Dark Triad and risk behaviors [35]. Lau and Marsee also found that narcissism showed the strongest associations with behavioral dysregulation and emotional dysregulation among the Dark Triad traits [43].

Another possibility for the obtained results is that sensation seeking is characteristically higher in men than that in women [44]. Regarding impulsivity and sensation seeking, the two key behaviors correlating to the fast life history strategy in humans are coupled with entitlement and overconfidence (i.e., narcissism), and men appear motivated to engage in reckless eating behaviors.

With respect to psychopathy, the present study found that secondary psychopathy was associated with higher uncontrolled eating behaviors in both sexes, and associated with higher emotional eating behaviors for men only. These results should be taken as modest support for our hypotheses, and allow for us to further discriminate secondary psychopathy from primary psychopathy. Previous research has revealed that, compared to primary psychopathy (emotionally stable psychopathy), secondary psychopathy (neurotic psychopathy) is a better predictor of uncontrolled behaviors such as substance abuse, aggression, and criminality [45]. Therefore, from a mental health perspective, skills in emotion regulation should be included when reckless eating behaviors are the focus of an intervention program.

Incidentally, the current study noted that those men who scored higher on the Dark Triad, especially narcissism, showed more reckless eating behaviors than their women counterparts did, while others found that eating disorders occur almost exclusively in females [3]. The solution of the apparent contradictory findings appears to be associated with the hypothesis that, as a female strategy in mating and status competition, women’s dieting behaviors are fundamentally adaptive and may lead to maladaptive outcomes, such as anorexia nervosa [1].

There are some limitations of the current study that should be considered. First, only self-report measures were used; therefore, the present study may be subject to monoinformant biases. Future studies may benefit from additional data sources, such as parents, teachers, peers, and close friends. Second, although Machiavellianism appears to be one-dimensional, both narcissism and psychopathy are multidimensional [46,47]. In this study, one characteristic limitation is that it tended to consider overall scores on the narcissism trait. Future research may examine the associations between eating behaviors and different types of narcissism, that is, grandiose and vulnerable narcissism. Third, while the study was available online, recruitment was reliant on Chinese-speaking populations. The culture from which participants are recruited may impact on personality, eating behaviors, and their willingness to provide socially desirable responses [48]. Future research should consider a more diverse population such as Western populations. In addition, the current work is based on a small sample of participants, so future researchers should attempt to replicate these findings in larger samples to gain a more reliable result. Fourth, although it is important to study eating behaviors in a subclinical sample, it limits the generalizability of the results to a population from clinical samples. Future research should extend it to clinical sample in order to determine whether similar associations between Dark Triad traits and eating behaviors emerge. Lastly, although the reliability and validity of the eating questionnaire in this study were satisfactory, the questionnaire had not been validated in the Chinese context before, and its psychometric properties need to be further tested in the future.

## 5. Conclusions

This study is explorational research to examine the associations between Dark Triad traits and eating behaviors. The results showed that (a) despite expected sex differences in narcissism and primary psychopathy, no sex differences were found in Machiavellianism, secondary psychopathy, and eating behaviors; (b) among women, Machiavellianism was a protective factor against uncontrolled eating behaviors; (c) the sex of the participant moderated the narcissism–uncontrolled eating and narcissism–emotional eating relationships, with the negative correlation being stronger for men than that for women; (d) secondary psychopathy, rather than primary psychopathy, was associated with higher uncontrolled eating behaviors in both sexes, and associated with higher emotional eating behaviors for men only.

## Figures and Tables

**Table 1:** Means and standard deviations by sex for all variables.

Domain	Measure	Men	Women	*t*
Dark Triad	Machiavellianism	43.646 ± 10.697	42.883 ± 9.268	0.690
	Narcissism	6.584 ± 4.356	5.129 ± 3.532	3.366 **
	Primary psychopathy	34.885 ± 7.309	32.976 ± 6.212	2.559 *
	Secondary psychopathy	21.938 ± 4.627	21.351 ± 4.154	1.201
Eating behaviors	Restrained eating	19.124 ± 4.753	18.710 ± 4.736	0.770
	Uncontrolled eating	24.735 ± 6.606	25.194 ± 5.492	−0.690
	Emotional eating	8.363 ± 2.781	8.218 ± 2.218	0.504

* *p* < 0.05, ** *p* < 0.01.

**Table 2:** Correlations between Dark Triad and all variables by sex.

	Restrained Eating				Uncontrolled Eating				Emotional Eating			
	Total	Men	Women	z	Total	Men	Women	z	Total	Men	Women	z
Machiavellianism	0.054	−0.011	0.087	−0.664	−0.002	0.052	−0.034	0.157	−0.006	0.018	−0.022	−0.035
Narcissism	−0.078	−0.151	−0.051	0.881	−0.211 **	−0.389 **	−0.087	2.818 **	−0.120 *	−0.308 **	−0.011	2.678 **
P psychopathy	−0.057	−0.137	−0.024	0.992	−0.203 **	−0.199 *	−0.200 **	−0.009	−0.114 *	−0.109	−0.124	−0.132
S psychopathy	−0.018	−0.093	0.016	0.673	−0.219 **	−0.192 *	−0.233 **	−0.374	−0.137 **	−0.172	−0.120	0.463

* *p* < 0.05, ** *p* < 0.01

**Table 3:** Multiple regression of Dark Triad traits on restrained eating by sex.

Variable	Total			Men			Women		
	*B*	*SE B*	*β*	*B*	*SE B*	*β*	*B*	*SE B*	*β*
Step 1									
Age	−0.045	0.033	−0.071	−0.115	0.052	−0.207 *	0.001	0.044	0.002
Educational degree	−0.729	0.395	−0.097	−0.503	0.694	−0.068	−0.734	0.488	−0.096
Step 2									
Age	−0.045	0.033	−0.070	−0.101	0.052	−0.183	−0.002	0.044	−0.003
Educational degree	−0.834	0.398	−0.111 *	−0.739	0.718	−0.099	−0.768	0.492	−0.100
Machiavellianism	0.074	0.034	0.151 *	0.052	0.060	0.117	0.083	0.042	0.163 *
Narcissism	−0.067	0.067	−0.055	−0.122	0.108	−0.112	−0.049	0.089	−0.036
P psychopathy	−0.091	0.053	−0.127	−0.096	0.087	−0.147	−0.084	0.069	−0.110
S psychopathy	−0.041	0.076	−0.038	−0.089	0.126	−0.087	−0.010	0.096	−0.009
Step 1 *R*²	0.015			0.051			0.009		
Step 2 *R*²	0.037			0.092			0.028		
Full model *F*	2.231 *			1.791			1.155		

* *p* < 0.05, ** *p* < 0.01.

**Table 4:** Multiple regression of Dark Triad traits on uncontrolled eating by sex.

Variable	Total			Men			Women		
	*B*	*SE B*	*β*	*B*	*SE B*	*β*	*B*	*SE B*	*β*
Step 1									
Age	0.069	0.041	0.088	−0.070	0.073	−0.091	0.161	0.050	0.201 **
Educational degree	0.293	0.489	0.032	1.583	0.976	0.153	−0.298	0.557	−0.034
Step 2									
Age	0.071	0.039	0.090	−0.031	0.066	−0.040	0.146	0.049	0.183 **
Educational degree	0.006	0.464	0.001	1.055	0.908	0.102	−0.414	0.541	−0.047
Machiavellianism	0.144	0.039	0.240 **	0.142	0.076	0.230	0.117	0.046	0.197 *
Narcissism	−0.303	0.078	−0.200 **	−0.553	0.137	−0.365 **	−0.119	0.098	−0.077
P psychopathy	−0.130	0.062	−0.146 *	−0.115	0.110	−0.128	−0.114	0.076	−0.128
S psychopathy	−0.377	0.089	−0.277 **	−0.384	0.159	−0.269 *	−0.341	0.106	−0.258 **
Step 1 *R*²	0.009			0.029			0.042		
Step 2 *R*²	0.140			0.249			0.124		
Full model *F*	9.587 **			5.849 **			5.674 **		

* *p* < 0.05, ** *p* < 0.01.

**Table 5:** Multiple regression of Dark Triad traits on emotional eating by sex.

Variable	Total			Men			Women		
	*B*	*SE B*	*β*	*B*	*SE B*	*β*	*B*	*SE B*	*β*
Step 1									
Age	0.036	0.018	0.105 *	−0.008	0.031	−0.024	0.062	0.022	0.177 **
Educational degree	−0.243	0.211	−0.061	0.372	0.415	0.086	−0.497	0.244	−0.127 *
Step 2									
Age	0.037	0.018	0.108 *	0.004	0.030	0.013	0.060	0.022	0.170 **
Educational degree	−0.324	0.210	−0.081	0.239	0.406	0.055	−0.550	0.245	−0.141 *
Machiavellianism	0.039	0.018	0.148 *	0.029	0.034	0.113	0.031	0.021	0.117
Narcissism	−0.073	0.035	−0.112 *	−0.206	0.061	−0.322 **	0.014	0.044	0.021
P psychopathy	−0.028	0.028	−0.073	0.009	0.049	0.024	−0.043	0.034	−0.111
S psychopathy	−0.112	0.040	−0.190 **	−0.165	0.071	−0.274 *	−0.065	0.048	−0.111
Step 1 *R*²	0.014			0.007			0.048		
Step 2 *R*²	0.063			0.150			0.073		
Full model *F*	3.933 **			3.125 **			3.129 **		

* *p* < 0.05, ** *p* < 0.01.

## Data Availability

The data presented in this study are available on request from the corresponding author.
